# Supplementary material for: Bayesian refinement of protein structures and ensembles against SAXS data using molecular dynamics
Source: PLoS Comput Biol. 2017 Oct 18;13(10):e1005800. doi: 10.1371/journal.pcbi.1005800 (PMC5662244; doi:10.1371/journal.pcbi.1005800)
Supplement: S1 Archive — (BZ2) [file pcbi.1005800.s011.tar.bz2 › gromacs_4.62_bayesian_saxs_refi/share/html/online/top.html]

top file format


## top file format

|  |  |
| --- | --- |
| Main Table of Contents | **VERSION 4.6** |
| GROMACS homepage | **Sat 19 Jan 2013** |

---

### Description

The top file extension stands for topology. It is an ascii file which is
read by grompp which processes it
and creates a binary topology (tpb-file).  
A sample file is included below:

```
;
;	Example topology file
;
[ defaults ]
; nbfunc        comb-rule       gen-pairs       fudgeLJ fudgeQQ
  1             1               no              1.0     1.0

; The force field files to be included
#include "rt41c5.itp"	

[ moleculetype ]
; name  nrexcl
Urea         3

[ atoms ]
;   nr    type   resnr  residu    atom    cgnr  charge
     1       C       1    UREA      C1       1	 0.683	
     2       O       1    UREA      O2       1	-0.683
     3      NT       1    UREA      N3       2	-0.622
     4       H       1    UREA      H4       2	 0.346
     5       H       1    UREA      H5       2	 0.276
     6      NT       1    UREA      N6       3	-0.622
     7       H       1    UREA      H7       3   0.346
     8       H       1    UREA      H8       3	 0.276

[ bonds ]
;  ai    aj funct           c0           c1
    3     4     1 1.000000e-01 3.744680e+05 
    3     5     1 1.000000e-01 3.744680e+05 
    6     7     1 1.000000e-01 3.744680e+05 
    6     8     1 1.000000e-01 3.744680e+05 
    1     2     1 1.230000e-01 5.020800e+05 
    1     3     1 1.330000e-01 3.765600e+05 
    1     6     1 1.330000e-01 3.765600e+05 

[ pairs ]
;  ai    aj funct           c0           c1
    2     4     1 0.000000e+00 0.000000e+00 
    2     5     1 0.000000e+00 0.000000e+00 
    2     7     1 0.000000e+00 0.000000e+00 
    2     8     1 0.000000e+00 0.000000e+00 
    3     7     1 0.000000e+00 0.000000e+00 
    3     8     1 0.000000e+00 0.000000e+00 
    4     6     1 0.000000e+00 0.000000e+00 
    5     6     1 0.000000e+00 0.000000e+00 

[ angles ]
;  ai    aj    ak funct           c0           c1
    1     3     4     1 1.200000e+02 2.928800e+02 
    1     3     5     1 1.200000e+02 2.928800e+02 
    4     3     5     1 1.200000e+02 3.347200e+02 
    1     6     7     1 1.200000e+02 2.928800e+02 
    1     6     8     1 1.200000e+02 2.928800e+02 
    7     6     8     1 1.200000e+02 3.347200e+02 
    2     1     3     1 1.215000e+02 5.020800e+02 
    2     1     6     1 1.215000e+02 5.020800e+02 
    3     1     6     1 1.170000e+02 5.020800e+02 

[ dihedrals ]
;  ai    aj    ak    al funct           c0           c1           c2
    2     1     3     4     1 1.800000e+02 3.347200e+01 2.000000e+00 
    6     1     3     4     1 1.800000e+02 3.347200e+01 2.000000e+00 
    2     1     3     5     1 1.800000e+02 3.347200e+01 2.000000e+00 
    6     1     3     5     1 1.800000e+02 3.347200e+01 2.000000e+00 
    2     1     6     7     1 1.800000e+02 3.347200e+01 2.000000e+00 
    3     1     6     7     1 1.800000e+02 3.347200e+01 2.000000e+00 
    2     1     6     8     1 1.800000e+02 3.347200e+01 2.000000e+00 
    3     1     6     8     1 1.800000e+02 3.347200e+01 2.000000e+00 

[ dihedrals ]
;  ai    aj    ak    al funct           c0           c1
    3     4     5     1     2 0.000000e+00 1.673600e+02 
    6     7     8     1     2 0.000000e+00 1.673600e+02 
    1     3     6     2     2 0.000000e+00 1.673600e+02 

; Include SPC water topology
#include "spc.itp"

[ system ]
Urea in Water

[ molecules ]
Urea	1
SOL	1000
```

---

http://www.gromacs.org
